# Gynaecology Teaching Associates in Medical Education—A Scoping Review

**DOI:** 10.1111/tct.70460

**Published:** 2026-06-10

**Authors:** Caitlin Kelly, Emily Bell, Megan E. L. Brown, Heidi Stelling

**Affiliations:** ^1^ School of Medicine Newcastle University Newcastle Upon Tyne UK

**Keywords:** GTA'S, Gynaecology Teaching Associate, pelvic examination, scoping review

## Abstract

**Background:**

Pelvic examination is a core, yet anxiety‐provoking, clinical skill in undergraduate medical education. Traditional teaching approaches—often opportunistic or reliant on simulation—have been criticised for disembodying technical skill from relational and ethical practice. Gynaecology Teaching Associates (GTAs), trained educators who use their own bodies to teach and provide embodied feedback, have emerged as an alternative model. This scoping review aimed to map what is known about GTAs, whose perspectives are represented, and what factors influence implementation.

**Methods:**

A scoping review was conducted in accordance with Arksey and O'Malley's framework and prospectively registered. Searches of MEDLINE, Embase, PsycINFO, ERIC and Scopus (June 2025) were supplemented by citation tracking and grey literature searches. English‐language sources focused on GTAs were included, with no date restriction. Screening and data extraction were undertaken by two reviewers, with thematic synthesis conducted inductively.

**Findings:**

Eighty‐three sources were included. Four interrelated domains were identified: educational outcomes, finance, ethics and representation/standardisation. GTA programmes are consistently associated with reduced student anxiety, improved confidence and enhanced communication skills, though evidence is frequently short‐term and self‐reported. Financial constraints and logistical complexity present ongoing barriers. Ethically, GTAs are positioned as an advance on historical practices, yet concerns regarding GTA well‐being and labour persist. Standardisation improves programme quality but may inadvertently narrow representations of bodily diversity.

**Implications:**

GTAs represent a pedagogically and ethically significant approach to pelvic examination teaching. Future research should prioritise longitudinal, multi‐institutional evaluation, clearer definitions of effectiveness, patient‐centred outcomes and co‐produced standards to support sustainable, equitable implementation.

## 1 Background

Pelvic examination is a core clinical skill and a key component of undergraduate medical education [1]. Competent performance of examination requires technical precision, anatomical knowledge and the ability to communicate clearly, sensitively and carefully [1]. Although technical and communication skills are inseparable in the practice of pelvic examination, undergraduate medical education often focuses on the stepwise teaching of pelvic examination as a disembodied process, without equal attention to relational and ethical dimensions of intimate physical examination [2].

Evidence consistently suggests that medical graduates feel underprepared to perform pelvic examinations, reporting low confidence and limited experience entering clinical practice [3]. This is a consequence of reduced exposure during training, variable supervision and inequitable access to learning opportunities, particularly for male students and those training in academic centres [4, 5]. Issues in early training have been linked to avoidance behaviours and reluctance to perform pelvic examinations independently, even beyond graduation [6]; negatively impacting patient care [3].

*Evidence consistently suggests that medical graduates feel underprepared to perform pelvic examinations, reporting low confidence and limited experience entering clinical practice*.

Intimate examinations are frequently associated with embarrassment, distress and loss of control for patients [7]. Concerningly, negative experiences of pelvic examination have been reported to negatively influence future engagement with gynaecological care, including attendance for cervical screening [8]. Qualitative studies highlight how poor communication, perceived detachment and lack of sensitivity during pelvic examinations contribute to mistrust and disengagement [9].

Historically, the teaching of pelvic examination relied on opportunistic learning during clinical placements or examinations performed under anaesthesia. The latter approach has been fiercely criticised, as explicit consent for educational examination under anaesthetic was often not obtained from women [10]. Further, practising examination under anaesthetic means appropriate communication skills cannot be rehearsed and refined by learners [11]. Alternative approaches to teaching, including the use of Gynaecology Teaching Associates (GTAs), emerged in response.

GTAs are trained educators who use their own bodies to teach pelvic examination skills while providing immediate, embodied feedback on technical performance and communication [12]. Unlike standardised patients, whose role is typically to portray a scenario for assessment or observation, GTAs function simultaneously as teacher, examiner and patient [13]. In a recent systematic review of approaches to improving medical student comfort and confidence in performing pelvic examinations, Kirabarajan et al. highlight GTAs as the most effective [3]. More broadly, evidence suggests that involving patients in the education of intimate examinations (including, but not limited to, pelvic examination) improves outcomes for learners in terms of confidence and performance [14]. When compared with education using pelvic models, GTA‐based education improves the examiner‐assessed competence of medical students and has been calculated as a cost‐effective approach to medical education [15, 16]. Yet, despite positive evidence regarding the potential impacts of GTAs, in 2018, only six UK medical schools employed GTAs within their curricula [17].

*GTAs are trained educators who use their own bodies to teach pelvic examination skills while providing immediate, embodied feedback on technical performance and communication*.

The existing research on GTAs is heterogenous in that it is fragmented across clinical, educational and ethical literatures, and spans several decades. Although reviews on GTAs exist, they are aging, and have tended to be systematic in nature, focused on discerning improvements in medical student performance outcomes. There has been little contemporary effort to map existing literature on GTAs and their implementation, including how knowledge has been produced and where conceptual and empirical gaps remain. Without mapping existing evidence in this way, it is difficult to understand what is actually known about GTAs, how they are positioned within medical education and what implications this has for curricula design and future research.

*There has been little contemporary effort to map existing literature on GTAs and their implementation, including how knowledge has been produced and where conceptual and empirical gaps remain*.

The aim of this scoping review, therefore, is to map existing literature on GTAs guided by the following research questions: What is known in the current literature about GTAs?, Whose perspectives are explored and represented in the existing literature on GTAs? and What barriers and enabler to implementing GTA‐led teaching in medical schools are identified in the literature?

## 2 Methods

This scoping review was conducted in accordance with the methodological framework developed by Arksey and O'Malley [18]. The review was prospectively registered with the Open Science Framework (OSF; https://doi.org/10.17605/OSF.IO/JEHR7) and is reported in accordance with the PRISMA‐ScR checklist (Data S1). Scoping review methodology was selected to allow a comprehensive and flexible approach to mapping diverse literature and because it is well‐suited to identifying research gaps.

### 2.1 Search Strategy

The search strategy was developed using the PCC (population–concept–context) framework and informed by team expertise. It was tailored to each database with the assistance of an academic medical librarian. Subject headings, free‐text word terms and synonyms relating to GTAs were included. The full search strategy is provided in Data S2. Searches were run in June 2025 across MEDLINE, Embase, PsycINFO (all via Ovid), ERIC (via EBSCOhost) and Scopus. Backward and forward citation tracking were undertaken to identify additional relevant studies. Grey literature searching and citation tracking were also undertaken.

### 2.2 Eligibility Criteria

Sources relating to GTAs (or synonymous terms) were eligible for inclusion, with no restriction on population, context, geographical location or study design. To allow exploration of developments and shifts in practice over time, no date limits were applied. Only sources published in English were included. While the search strategy was intentionally broad in scope, restrictions were applied by publication type: Conference abstracts, opinion pieces, editorials and letters were excluded.

### 2.3 Screening and Data Extraction

Search results were imported into Zotero and screened using Rayyan. Screening was undertaken by two reviewers (CK/EB) against predetermined eligibility criteria. A random 10% sample was independently double‐screened (HS), and any discrepancies were resolved through discussion with the wider review team. A structured data extraction tool was piloted and refined before full extraction. Thematic synthesis was conducted inductively.

### 2.4 Reflexivity

Reflexivity was embedded throughout. Reviewers (CK/EB) kept reflexive diaries through screening, discussed in weekly team meetings. Double screening enabled reviewers to reflect on how previous experiences shaped decision‐making and to make these explicit through shared reflection.

## 3 Findings

### 3.1 Characteristics of Included Studies

Searches retrieved 1261 sources. Screening identified 83 sources included in the final review. The screening process is summarised in the PRISMA flowchart (Data S3).

The prefix ‘G’ has been added to all included references to enable cross‐checking with Tables 1 and 2 and Data S4 which includes a full summary of sources.

**TABLE 1:** Summary of positive educational outcomes associated with GTA programmes.

Domain | number of papers | references	
Reduced anxiety and improved confidence	
28	G1, G50, G51, G15, G56, G58, G60, G64, G72, G73, G21, G81, G24, G82, G25, G61, G75, G39, G49, G28, G23, G62, G34, G36, G66, G3, G16, G38
Enhanced communication and professional behaviours	
19	G46, G47, G6, G51, G55, G65, G69, G73, G21, G81, G25, G62, G42, G43, G45, G22, G74, G53, G27
Increased skill, knowledge and competence	
27	G46, G47, G55, G56, G9, G17, G65, G70, G72, G73, G81, G82, G75, G49, G28, G31, G23, G62, G35, G36, G41, G43, G22, G19, G66, G2, G27
Improved learning environment	
13	G59, G9, G15, G64, G65, G70, G71, G82, G30, G75, G49, G5, G66
Increased willingness to seek patient examination opportunities	
2	G56, G65
Standardised medical student opportunities	
6	G15, G24, G34, G21, G19, G54

**TABLE 2:** Barriers and enablers influencing the implementation of GTA programmes by factor.

Factor	Barriers/challenges number of papers | references		Enablers/supporters number of papers | references	
Financial considerations and cost‐effectiveness	Short‐term financial costs, unclear funding responsibilities, and administrative pressures are key barriers to GTA programme implementation.		Long‐term cost‐effectiveness can be achieved through reduced faculty involvement, improved student outcomes, and operational efficiencies, making GTA programmes a worthwhile investment in some contexts.	
	9	G7, G57, G18, G72, G82, G35, G36, G37, G16	9	G46, G51, G58, G80, G30, G35, G41, G42, G44
Recruitment and workforce sustainability	Recruitment and retention are constrained by a small, highly specific candidate pool, time‐intensive training requirements, increasing student numbers and the need to ensure consistent quality, safety, and welfare.		Recruitment and retention can be facilitated by values‐driven candidates with strong interest in women's health, alternative recruitment pools, feasible training models and settings where GTAs demonstrate long‐term commitment.	
	11	G6, G47, G59, G72, G79, G81, G82, G39, G62, G34, G66	6	G55, G20, G40, G42, G44, G76
Training logistics and hybrid teaching approaches	Implementation of GTA programmes is constrained by time‐intensive training, small‐group requirements, limited practice opportunities, supervision needs, timetabling challenges and cultural or curricular barriers.		Hybrid models combining GTA and manikin‐based teaching enhance feasibility, improve confidence, reduce anxiety and optimise clinical skills while mitigating logistical constraints.	
	14	G47, G56, G59, G63, G18, G21, G71, G72, G81, G82, G39, G62, G36, G53	3	G63, G61, G33
Ethics, autonomy, and GTA well‐being	Student discomfort and GTA well‐being concerns—including physical strain, emotional stress and stigma—pose significant ethical and practical barriers to implementing GTA programmes		GTA programmes promote ethical practice and safeguard well‐being by teaching respect for patient autonomy, modelling professional boundaries and providing structured support that reduces discomfort and fosters GTA confidence and contribution	
	12	G67, G78, G8, G20, G81, G26, G31, G13, G74, G68, G77, G4	11	G8, G20, G39, G26, G33, G6, G14, G57, G72, G68, G32
Evidence base and long‐term effectiveness	Limited and variable evidence, single‐centre studies and inconsistent GTA practices across institutions constrain generalisability, transferability and justification for programme investment.		Some follow‐up studies and graduate feedback suggest sustained perceived benefits, but evidence remains limited, particularly for real‐patient outcomes and multicentre, long‐term evaluation	
	14	G47, G18, G64, G65, G69, G70, G71, G72, G78, G28, G62, G17, G29, G66	2	G46, G56
Direct feedback: educational benefits and assessment implications	Involving GTAs in the assessment of students they have taught risks evaluator bias, potentially inflating performance scores and overstating short‐term educational gains.		Immediate, embodied feedback from GTAs in a supportive, low‐stakes environment enhances technical competence, communication skills, confidence and preparedness for real‐patient encounters.	
	4	G47, G21, G25, G75	21	G47, G51, G9, G15, G59, G65, G70, G71, G78, G79, G25, G24, G81, G82, G30, G61, G39, G28, G23, G45, G66

Included studies were heavily skewed towards the Global North. Most originated from North America (*n* = 44) and Europe (*n* = 33), with smaller numbers from Oceania (*n* = 9) and Asia (*n* = 2). Publication dates ranged from 1975 to 2023. However, output has increased substantially over time: 58 papers (approximately 70% of the total sample) were published in the 25 years from 2000 onwards, compared with 25 in the preceding 25‐year period. This trend suggests growing scholarly attention to pelvic examination education.

Across included studies, the perspectives of GTAs, students and faculty were variably represented, with GTA and student voices more prominent than those of educators and the patient voice notably absent. Student and GTA perspectives were resoundingly positive, frequently emphasising psychological safety, authenticity of learning and the value of embodied, experiential expertise. Faculty views were more mixed, often supportive of the educational benefits but tempered by concerns regarding cost, logistical complexity, sustainability and institutional resource allocation. The absence of direct patient perspectives is striking, particularly given that improved patient experience and safety are commonly invoked as key justifications for GTA programmes.

*Across included studies, the perspectives of GTAs, students and faculty were variably represented, with GTA and student voices more prominent than those of educators and the patient voice notably absent*.

### 3.2 Overview of Findings

**FIGURE 1:**
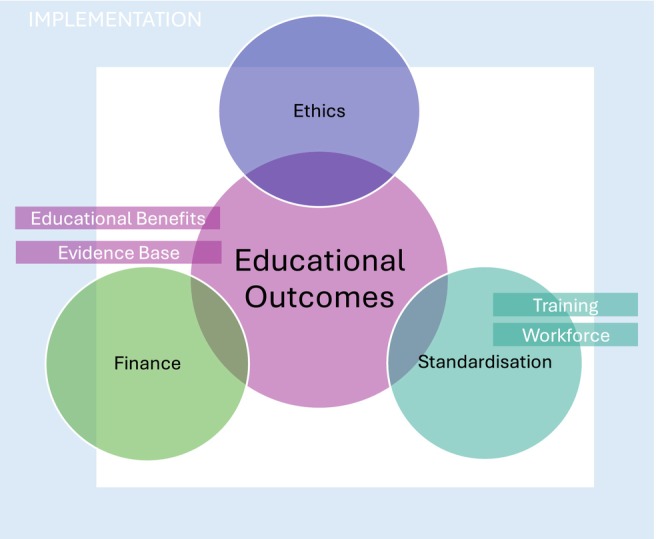
Conceptual framework illustrating the four domains underpinning GTAs, how they interact and influence programme implementation. The central circle represents the GTA literature in sum, with educational outcomes positioned at its core and intersecting with the three surrounding domains: ethics, finance and standardisation. The outer box represents factors directly influencing implementation. Ethics and finance extend most explicitly into this implementation space, reflecting their immediate structural and institutional implications. Subcomponents of educational outcomes and standardisation similarly overlap into the implementation domain, illustrating how pedagogical design, assessment practices, and workforce structures shape the feasibility and sustainability of GTA programmes in practice.

In line with our research questions, these findings synthesise what is known in the literature about GTA programmes across four interrelated domains: educational outcomes, finance, ethical considerations and representation and standardisation. How these domains impact GTA programme implementation is expanded upon in each section and summarised in Table 2, with Figure 1 bringing the findings together conceptually.

#### 3.2.1 Educational Outcomes

Educational outcomes are the most extensively reported domain in the GTA literature, with 55 papers addressing positive impacts on student learning and experience (Table 1). GTA programmes consistently reduce student anxiety and improve confidence, with learners reporting feeling calmer, more secure and better prepared for pelvic examinations following sessions (G1, G50, G51, G15, G56, G60, G82, G34, G16, G38). Confidence increased more for male students (G60) and was linked to previous sexual experience in both sexes (G1). While a few studies found no statistically significant differences compared to alternative teaching methods (G73, G24), overall, evidence supports a positive effect of GTA practice on reducing anxiety and enhancing confidence (G21, G36, G66).

*GTA programmes consistently reduce student anxiety and improve confidence, with learners reporting feeling calmer, more secure and better prepared for pelvic examinations following sessions*.

GTA involvement also enhances communication, professionalism and interpersonal skills. Students demonstrate greater awareness of patient comfort, autonomy and empathy. Further, structured GTA feedback improves supportive communication behaviours, including reassurance, rapport‐building and attention to patient anxiety (G46, G47, G6, G51, G55, G21, G81, G25, G45, G53, G66). Although some long‐term studies report no sustained differences compared to non‐GTA groups (G46, G81), immediate benefits for patient‐centred communication and professional development are consistently observed.

Across 27 studies, GTA training is associated with improvements in technical skill, knowledge and overall competence (Table 1). GTA‐trained students outperform peers using manikins or clinical patients in pelvic examination technique, procedural sequencing, psychomotor skills and comfort during procedures (G65). Diagnostic ability, however, does not appear to differ from manikin‐trained students (G65).

GTAs facilitate a structured, supportive learning environment with immediate feedback and repeated practice, improving student comfort, willingness to seek clinical opportunities and preparedness for real‐world placements (G56, G65, G19). They also help address gendered disparities in access to pelvic examination experience, standardising opportunities (G34, G15, G24). Furthermore, GTA sessions enable formal and comprehensive assessment of competence where real‐patient evaluation would otherwise be difficult (G37).

Evidence for improved OSCE performance is mixed, with some studies showing no difference between GTA‐trained students and those taught by faculty, manikins or standardised patients (G58, G29). Many studies rely on self‐reported measures such as confidence, anxiety or perceived competence (G1, G7, G8, G58, G60, G70, G71, G73, G78, G21, G82, G61, G49, G28, G66). Additionally, isolating GTA effects from concurrent clinical experiences is challenging, as independent study or clerkship opportunities may influence outcomes (G30, G29). A systematic review found no evidence of direct patient benefit from GTA use in medical education (G43); however, the literature also identifies a lack of large‐scale studies with standardised, measurable outcomes capable of evaluating patient‐level impact (G73, G80).

Limited and variable evidence regarding educational outcomes raise concerns about generalisability and long‐term impact, posing barriers to implementation (Table 2). Studies are often single‐centre, with small samples and wide variation in GTA use, timing and integration across institutions (G47, G18, G69, G28, G62, G66). Long‐term data are sparse, and some studies report no sustained skill advantage compared with manikin‐trained students (G17, G29). Nonetheless, graduate feedback indicates enduring support for GTA programmes, suggesting perceived value despite limited empirical data (G46, G56). Gaps in the literature include limited evidence on long‐term patient outcomes, uncertainty regarding optimal delivery and a lack of multicentre longitudinal data across differing cultural and ethical contexts. These gaps constrain evidence available to support implementation (G58).

Real‐time, embodied feedback emerges as the key driver of GTA utility (Table 2). GTAs guide students' hands, confirm anatomy and signal discomfort within a supportive environment, enhancing technical competence, interpersonal sensitivity and confidence (G51, G47, G78, G25, G9, G24, G15, G45, G3). However, when GTAs assess students they have taught, familiarity may bias evaluation (Table 2), potentially inflating scores and limiting sustained skill acquisition (G47, G21, G25, G46). Tensions between the pedagogical value of direct GTA feedback and concerns about assessment validity when teaching and evaluation roles overlap have been reported (G75).

#### 3.2.2 Finance

Women's health training, including GTA programmes, may be vulnerable to de‐prioritisation, with reviews of GTA implementation describing variable uptake and sustainability within resource‐constrained curricula (G31, G35). GTA programmes require significant upfront and ongoing investment, creating short‐term funding challenges for medical schools (G6, G18, G72, G16). Ambiguity over whether funding responsibility lies with medical schools or departments complicates planning (G7). Economic evaluations indicate higher per‐student costs, increased operational and administrative burden and limited evidence of long‐term clinical benefit, which collectively restrict scalability and sustainability (G35, G36). However, longitudinal economic evaluation suggests acceptable cost‐effectiveness when benefits are considered across a doctor's career trajectory (G35).

Faculty involvement also affects financial and logistical considerations. While some studies report no significant difference in outcomes when GTAs teach without faculty, others note student unease in the absence of a clinician (G58, G21, G23). Removing faculty from sessions improves cost‐effectiveness and reduces logistical burden while maintaining educational outcomes (G30, G80), although institutional acceptability and learner reassurance must be considered (G60, G52).

Financial demands and budgetary constraints remain a key barrier to GTA implementation (Table 2) despite strong perceived educational value (G36, G37, G18). All six UK medical schools currently employing GTAs reported their programmes to be educationally successful; however, funding limitations restricted delivery, with most institutions able to provide only a single GTA exposure per student (G37).

*Financial demands and budgetary constraints remain a key barrier to GTA implementation (Table* *2* *) despite strong perceived educational value*.

At the same time, multiple studies justify continued investment in GTA programmes on the basis of their educational benefits and potential cost‐effectiveness when appropriately structured (G46, G51, G58, G80, G30, G35, G41, G42, G44). Models delivered without direct faculty involvement were associated with reduced costs and more efficient use of clinician time, suggesting opportunities to reallocate faculty effort while maintaining educational quality (G30). Evidence from COVID‐19‐related curricula disruption showed that removal of GTA teaching led to reduced student confidence in pelvic examinations, reinforcing the value of institutional investment (G41). Historical and contemporary evidence suggests GTA programmes can be cost‐effective when scaled through efficient scheduling and increased GTA availability, with further gains possible by expanding GTA roles beyond pelvic examination teaching (G42, G44, G11).

#### 3.2.3 Ethical Considerations

Ethical considerations are central in the literature. GTA programmes are both a source of concern and seen as deliberate improvement on traditional teaching. Initial faculty scepticism reflected the novelty of the approach (G47, G18), while GTA well‐being emerged as a key concern. GTAs navigate complex roles as teachers, patients and professionals, exposing them to physical strain, fatigue and emotional labour which may affect teaching quality and assessment (G75, G26).

Despite challenges, GTA programmes are consistently framed as more ethical than traditional methods. They provide a consent‐focused, respectful environment, modelling boundaries, dignity and professional communication, which reinforce the doctor–patient relationship and ethical practice (G59, G82, G72, G83, G12, G5, G68, G38). Historical evidence highlights the ethical imperative for this shift, documenting the use of anaesthetised or hospitalised women—and, in some settings, sex workers—as teaching resources. These practices have been increasingly challenged and replaced by alternative approaches including GTA models (G10, G12).

Barriers related to ethical concerns and GTA well‐being were widely reported (Table 2) and are key factors impacting implementation. Students sometimes fail to recognise GTAs as individuals with histories and emotions, with male students reporting vulnerability, embarrassment and discomfort during exams (G67, G78). GTAs experience physical strain, including vaginal soreness, pelvic pain and fatigue, alongside emotional burdens such as embarrassment, anxiety and feelings of being treated as a ‘tool’ (G8, G20, G81, G26, G13, G68, G77, G4). Limited adherence to safety frameworks and inconsistent protections further raises ethical concerns, including potential exploitation and stigma (G26, G31, G74).

Conversely, structured GTA programmes can safeguard well‐being and promote ethical practice. Teaching students about patient autonomy, gender and power dynamics, alongside clear role boundaries, structured scripts and paired teaching, provides GTAs with emotional and practical support. These measures enhance communication, assertiveness and a sense of contribution while reducing student‐caused discomfort (G6, G14, G8, G20, G39, G33, G26, G32).

#### 3.2.4 Representation and Standardisation

The diversity and lived experiences of GTAs enhance teaching credibility, foster empathy and support student understanding of medicine's gender and power dynamics (G50, G7, G26). However, institutional standardisation introduced to professionalise GTA programmes and ensure consistency in teaching, assessment, and conduct has also led to the standardisation of women's bodies within teaching contexts (G48, G42, G26). Several authors argue that this reduction in anatomical and experiential diversity may undermine the programme's original purpose, promoting a hidden curriculum of ‘normality’ and limiting student exposure to real‐world variation (G48, G26, G42).

In contrast, standardisation of pedagogical elements—such as GTA training, commitment, teaching approach and assessment practices—is consistently identified as important for programme quality and professionalism. Variation in GTA preparation and teaching ability influences learning outcomes, with structured training and supportive supervision described as critical enablers (G55, G60, G15, G73, G80, G49, G29, G44). Well‐prepared and committed GTAs are repeatedly identified as central to effective and valued learning experiences (G56, G59, G60, G23).

Recruitment and workforce sustainability are frequently cited implantation barriers (Table 2) due to the small, highly specific pool of candidates—women willing to undergo intimate examinations and able to teach effectively (G47, G6). Recruiting, training and maintaining a sufficiently sized GTA workforce is time‐intensive, particularly given rising student numbers and the need for small‐group teaching (G82, G79). Additional challenges include ensuring teaching consistency, an aging GTA population, cultural barriers and the need for robust oversight to safeguard GTA and student welfare (G62, G34, G77).

Conversely, well‐designed recruitment strategies can drive programme success. UK evidence demonstrates that GTA programmes can be successfully integrated into curricula, with women motivated by interest in women's health and meaningful, values‐driven work (G55, G20, G40). Retention is not always problematic, with GTAs often remaining beyond minimum commitments, and effective training can be achieved within relatively short timeframes (6–8 weeks) (G40, G42).

Training GTAs is resource‐intensive, requiring structured preparation to support quality (G47, G59, G53). Effective GTA teaching relies on small‐group formats, repeated exposure, adequate supervision and protected practice time; however, limited student opportunities, faculty availability, and physical space constrain scalability (G82, G39, G21). Timetabling and integration into crowded curricula, along with cultural sensitivities in some settings, further challenge implementation (G18, G62, G36).

Hybrid teaching models combining GTAs and manikin‐based simulation may optimise feasibility and learning outcomes. Evidence regarding sequencing is mixed: Early GTA exposure can enhance confidence and reduce anxiety (G63), while prior manikin practice may better prepare students for GTA sessions, improving technical skills, professionalism, and comfort during examinations (G33).

## 4 Implications

The aim of this scoping review was to map what is known about GTAs, whose perspectives are represented within available literature, and the factors shaping GTA implementation within undergraduate medical education.

Literature reporting the educational outcomes associated with use of GTAs is most heavily represented within this review. GTA programmes are repeatedly associated with reduced student anxiety, increased confidence and improvements in communication and interpersonal skills. This is important, given persistent evidence that pelvic examination causes anxiety for learners, and is seen as difficult to practise safely within opportunistic clinical settings [3]. Pedagogically, GTA teaching provides a structured environment in which technical skill can be developed in tandem with complex communication skills.

However, such a heavy emphasis on learner confidence and competence also reveals important limitations in how effectiveness has been conceptualised and evaluated within GTA literature. Many studies rely heavily on self‐reported outcomes and short‐term measures, reflective of broader challenges in medical education research [19]. Patient‐level outcomes, including experiences of care and longer term engagement with gynaecological services, appear largely absent from the evidence base. This is a notable gap, given that many educational justifications for GTA programmes are patient‐centred.

*However, such a heavy emphasis on learner confidence and competence also reveals important limitations in how effectiveness has been conceptualised and evaluated within GTA literature*.

Sociologically, GTAs appear to function as a corrective to the disembodiment that characterises much medical training [20]. Simulation approaches to education may provide standardisation and safety, but they abstract the body from its social and relational context [21]. In contrast, GTA teaching foregrounds the lived body as a site of learning, as it requires students to attend to consent, communication and bodily responsiveness in practice.

Financial considerations are reported as a persistent barrier to implementation, with GTA programmes frequently framed as resource‐intensive and difficult to sustain. Yet, cost‐effectiveness is highly sensitive to what outcomes are prioritised and over what timeframe value is assessed [22]. When educational benefits such as improved communication, reduced anxiety and preparedness for clinical practice are considered alongside potential efficiencies in faculty time, GTA programmes may represent a more nuanced investment than headline per‐student costs suggest [16]. The limited use of economic evaluation frameworks that capture these broader benefits within GTA literature constrains meaningful comparison with alternative teaching approaches.

Ethical considerations feature throughout available literature. GTA programmes are consistently positioned as an ethical advancement on historical teaching practices, particularly those involving examinations under anaesthesia without formal consent. At the same time, this review highlights ethical tensions related to GTA well‐being, emotional labour and bodily strain. Where programmes lack robust safeguards, GTAs may experience discomfort, fatigue or feel instrumentalised by the programme. It is important that ethics is viewed as also involving the conditions under which GTAs work and teach, as well as involving important issues of consent.

Concerns relating to representation and standardisation complicate implementation of GTAs. While standardisation is frequently invoked as a marker of quality and professionalism, several authors caution that excessive standardisation risks reproducing a narrow conception of the “normal” female body. This tension reflects a broader dilemma within medical education: The desire for fairness and comparability in assessment sits uneasily alongside uncertain, variable clinical and bodily realities [23]. GTA programmes, by their nature, expose students to embodied difference, and attempts to over‐regulate this exposure may undermine one of their core pedagogical strengths.

The review also highlights that medical student and educator perspectives are overrepresented within available literature. Patient perspectives are largely missing, despite frequent claims about patient‐centred benefits. GTA's voices, while present in some studies, remain inconsistently highlighted, particularly in relation to programme design and evaluation. These absences limit the capacity of the existing evidence base to inform socially responsible approaches to curricula design. More broadly, the marginalisation of patient perspectives may also reflect wider tendencies within medical education to privilege learner satisfaction, confidence and performance outcomes as markers of educational effectiveness. As Bleakley and colleagues argue, medical education has historically prioritised professional and institutional perspectives, often positioning patients more as educational resources than as active contributors to knowledge production and curriculum evaluation [24].

Taken together, the findings suggest that GTA programmes should be understood as part of broader educational strategy, rather than as a replacement for simulation or workplace‐based learning. Pelvic examination training appears most effective when multiple modalities are integrated across curricula, allowing students to develop technical skills, embodied awareness, and communication skills over time. GTA teaching offers particular value at points in training where anxiety is high and opportunities for supported practice are limited. This review also underscores the need for clearer guidance on programme design, including minimum standards for GTA training, support and safeguarding. Co‐production with GTAs may offer a way to address ethical concerns while strengthening programme sustainability. Institutions considering implementation should also be cautious about framing standardisation as an unqualified good, recognising that exposure to variation is integral to competent clinical practice.

*GTA teaching offers particular value at points in training where anxiety is high and opportunities for supported practice are limited*.

For future research, this review demonstrates a need to move beyond short‐term learner outcomes towards more theoretically informed and methodologically diverse evaluations, inclusive of patient and GTA perspectives. Longitudinal studies that examine how early training influences later clinical behaviour, patient experience and engagement with gynaecological care would strengthen existing evidence.

### 4.1 Directions for Future Research

There is a clear need to evaluate current practice nationally, mapping variation in delivery models, the use of GTAs and the educational, ethical, cost and representational implications of different approaches. Defining “effectiveness” is critical: Outcomes must extend beyond immediate learner confidence or skill acquisition to encompass longer term impacts on patient experience, safety and educator well‐being. In parallel, work is needed to develop nationally agreed standards for pelvic examination education that integrate ethical guidance, minimal exposure expectations for learners, GTA safety and well‐being frameworks and pedagogical rather than anatomical standardisation.

### 4.2 Strengths and Limitations

This review draws together literature on GTAs spanning educational, ethical and implementation research across several decades. By mapping how knowledge about GTAs has been produced and where gaps exist, it offers a foundation for future research and curricula development.

A key limitation is the inclusion of English‐language publications only, introducing potential language bias. While the breadth of included studies allowed for identification of cross‐cutting themes, this necessarily limited the depth with which local implementation challenges could be explored. As with all scoping reviews, the aim was to map the literature rather than appraise effectiveness, and so conclusions about impact should be interpreted cautiously.

## 5 Conclusion

The literature on GTAs presents a strikingly consistent message: GTA programmes improve learner confidence, reduce anxiety and enhance communication and technical skill in the context of an intimate and complex examination. Across decades of research, there is remarkably little evidence of harm and sustained acceptability among students and GTAs alike. Financial concerns, while frequently cited, are nuanced; when faculty time, scalability and potential long‐term value are considered, cost need not be prohibitive.

What remains absent is proof of longer term patient impact. Claims that GTA programmes improve patient experience, trust or engagement with gynaecological care—including cervical screening uptake—are attractive but empirically underexplored. If we wish to understand whether early, embodied, consent‐focused teaching shapes later clinical behaviour and patient outcomes, we must design research capable of capturing these trajectories. Establishing causation in complex systems will be challenging but is neither methodologically impossible nor ethically unjustified.

Taken together, GTA programmes appear consistent with current expectations around consent, dignity and patient‐centred care. Given the lack of evidence of harm and consistent short‐term educational benefits, the key issue is no longer whether GTAs work for learners but how we should evaluate longer term impact. Future research should examine whether these early educational gains translate into better patient care in clinical practice.

## Author Contributions

**Caitlin Kelly:** conceptualization, writing – review and editing, formal analysis. **Emily Bell:** conceptualization, writing – review and editing, formal analysis. **Megan E. L. Brown:** writing – review and editing, writing – original draft, formal analysis, supervision. **Heidi Stelling:** writing – review and editing, writing – original draft, funding acquisition, formal analysis, project administration, supervision.

## Funding

This project was supported through Newcastle University Careers Service Student Employment on Campus funding, which provided financial support for the student researchers (C.K., E.B.).

## Conflicts of Interest

The authors declare no conflicts of interest.

## Supporting information


**Data S1:** PRISMA Reporting Checklist.

**Data S2:** Final Search Strategy.

**Data S3:** PRISMA Flow Diagram.

**Data S4:** Summary of Included Studies.

## Data Availability

The datasets used and/or analysed during the current study are available from the corresponding author on reasonable request.
