# Supplementary material for: Gynaecology Teaching Associates in Medical Education—A Scoping Review
Source: Clin Teach. 2026 Jun 10;23(4):e70460. doi: 10.1111/tct.70460 (PMC13250820; doi:10.1111/tct.70460)
Supplement: Supplementary file 2 — Data S2: Final Search Strategy. [file TCT-23-e70460-s001.docx]

Supplementary File 4 - Summary of Included Sources

| **Ref.** | **Focus** | **Source Title** | **Authors' Name** | **Continent of Origin** | **Publication Year** | **Journal / Source** |
| --- | --- | --- | --- | --- | --- | --- |
| G1 | Educational outcomes | Anxiety and feelings of medical students conducting their first gynecological examination. | Abraham, S and Chapman, M and Taylor, A and McBride, A and Boyd, C | Oceania | 2003 | Journal of Psychosomatic Obstetrics & Gynecology |
| G2 | Educational outcomes (Table 1) | Gynaecological examination: a teaching package integrating assessment with learning. | Abraham, S. | Oceania | 1998 | Medical Education |
| G3 | Educational outcomes | An evaluation of the educational impact of Gynaecological Teaching Associates in teaching female pelvic examination. | Aisha Ayaz Janjua | Europe | 2017 | Univeristy of Birmingham |
| G4 | Ethical considerations | Made a career out of showing people my cervix' | Alexandra Duncan | North America | 2015 | BuzzFeed |
| G5 | Ethical considerations | Gynecological Teaching Associates. | Annie Oeth | North America | 2021 | University of Mississippi Medical Center (UMC) News |
| G6 | Educational outcomes Finance Ethical considerations Standardisation | Training gynaecology teaching associates | Beckmann C.R.B. and Barzansky B.M. and Sharf B.F. and Meyers K. | North America | 1988 | Medical Education |
| G7 | Educational outcomes Finance Standardisation | Gynaecological teaching associates in the 1990s. | Beckmann, C R and Lipscomb, G H and Williford, L and Bryant, E and Ling, F W | North America | 1992 | Medical education |
| G8 | Educational outcomes Ethical considerations | Mental and physical effects of being a gynecologic teaching associate. | Beckmann, C R and Meyers, K | North America | 1988 | The Journal of reproductive medicine |
| G9 | Educational outcomes | Student response to gynecologic teaching associates. | Beckmann, C R and Sharf, B F and Barzansky, B M and Spellacy, W N | North America | 1986 | American journal of obstetrics and gynecology |
| G10 | Ethical considerations | Initial instruction in the pelvic examination in the United States and Canada, 1983 | Beckmann, C.R., Spellacy, W.N., Yonke, A., Barzansky, B. and Cunningham, R.P. | North America | 1985 | American journal of obstetrics and gynecology |
| G11 | Finance | Dealing with sexual questions during consultations: A new training program. | Boendermaker, P. M and Faber, V and Schultz, W. C. M. Weijmar and Beckmann, C. R., Barzansky, B. M., Sharf, B. F., Meyers, K., Beckmann, Beckmann, C. R. B., Lipsco... | Europe | 2008 | Journal of Psychosomatic Obstetrics & Gynecology |
| G12 | Ethical considerations | Standardized patients and gynecological teaching associates. | Clark, L., Weaks, C., Dorsey, R.M., Strickland, V. and McAdam, S | North America | 2019 | In Comprehensive Healthcare Simulation: Obstetrics and Gynecology |
| G13 | Ethical considerations | Gynaecological teaching associates | Cowdrey, L | Europe | 2004 | BMJ |
| G14 | Ethical considerations | "What Do You Do if They Say 'No'?": Agency, Materiality, and Justice in Pelvic Exams. | Cusanno, Brianna R | Europe | 2023 | Health communication |
| G15 | Educational outcomes Standardisation | Medical students' experiences in learning to perform pelvic examinations: a mixed-methods study. | Danielsson, Johanna and Hadding, Cecilia and Fahlstrom, Martin and Ottander, Ulrika and Lindquist, David | Europe | 2021 | International journal of medical education |
| G16 | Educational outcomes Finance | Learning the breast examination with Physical Exam Teaching Associates: development and evaluation of the teaching setup. An action research approach. | den Harder, C., van den Eertwegh, V., Jongen, F., Hageraats, E., Nelissen, S. and Waterval, D. | Europe | 2022 | Women & Health |
| G17 | Educational outcomes | Educational effectiveness of gynaecological teaching associates: a multi-centre randomised controlled trial. | Duffy, J M N and Chequer, S and Braddy, A and Mylan, S and Royuela, A and Zamora, J and Ip, J and Hayden, S and Showell, M and Kinnersley, P and Chenoy, R and Westwood, O M and Khan, K S and Cushing, A | Europe | 2016 | BJOG : an international journal of obstetrics and gynaecology |
| G18 | Educational outcomes Finance Ethical considerations Standardisation | Pelvic and breast examination skills curricula in United States medical schools: a survey of obstetrics and gynecology clerkship directors. | Dugoff, Lorraine and Pradhan, Archana and Casey, Petra and Dalrymple, John L and Abbott, Jodi F and Buery-Joyner, Samantha D and Chuang, Alice and Cullimore, Amie J and Forstein, David A and Hampton, Brittany S and Kaczmarczyk, Joseph M and Katz, Nadine T and Nuthalapaty, Francis S and Page-Ramsey, Sarah M and Wolf, Abigail and Hueppchen, Nancy A | North America | 2016 | BMC medical education |
| G19 | Educational outcomes | To the point: the expanding role of simulation in obstetrics and gynecology medical student education. | Everett, E.N., Forstein, D.A., Bliss, S., Buery-Joyner, S.D., Craig, L.B., Graziano, S.C., Hampton, B.S., Hopkins, L., McKenzie, M.L., Morgan, H. and Pradhan, A | North America | 2019 | American journal of obstetrics and gynecology |
| G20 | Ethical considerations Standardisation | Women's experiences of working as a Clinical Teaching Associate teaching sensitive examinations: a qualitative study. | Fairbank, Christine and Reid, Katharine and Minzenmay, Krista | Europe | 2015 | Medical teacher |
| G21 | Educational outcomes Finance Standardisation | Evaluation of students' clinical and communication skills in performing a gynecologic examination. | Fang, Wei Li and Hillard, Paula J and Lindsay, Richard W and Underwood, Paul B | North America | 1984 | Journal of Medical Education |
| G22 | Educational outcomes (Table 1) | Differences in teaching female and male intimate examinations: A qualitative study. | Gleisner, Jenny and Siwe, Karin | Europe | 2020 | Medical education |
| G23 | Finance Standardisation | Can physicians be replaced with gynecological teaching women to train medical students in their first pelvic examination? A pilot study from Northern Sweden. | Grankvist, O., Olofsson, A.D. and Isaksson, R.M. | Europe | 2014 | Patient education and counseling |
| G24 | Educational outcomes | Effectiveness of the gynecology teaching associate in teaching pelvic examination skills. | Guenther, S M and Laube, D W and Matthes, S | North America | 1983 | Journal of medical education |
| G25 | Educational outcomes | Professional patients: an improved method of teaching breast and pelvic examination. | Hale, R W and Schiner, W | North America | 1977 | The Journal of reproductive medicine |
| G26 | Perspective Ethical considerations Standardisation | (per)forming the practice(d) body: Gynecological teaching associates in medical education | Hall, J. | North America | 2015 | Professional and Practice-based Learning |
| G27 | Educational outcomes (Table 1) | Use of Standardized Patients for Teaching and Evaluating the Genitourinary Examination Skills of Internal Medicine Residents | Hawkins, R. and Gross, R. and Beuttel, S. and Holmboe, E. and Gliva-McConvey, G. and Haley, H. | North America | 1998 | Teaching and Learning in Medicine |
| G28 | Educational outcomes | Learning intimate examinations with simulated patients: the evaluation of medical students’ performance. | Hendrickx, K., De Winter, B., Tjalma, W., Avonts, D., Peeraer, G. and Wyndaele, J.J | Europe | 2009 | Medical teacher |
| G29 | Educational outcomes Standardisation | Medical students' gynecologic examination skills. Evaluation by gynecology teaching associates. | Hillard, P J and Fang, W L | North America | 1986 | The Journal of reproductive medicine |
| G30 | Educational outcomes Finance | Initial pelvic examination Instruction: The Effectiveness of Three Contemporary Approaches. | Holzman, G. B., Singleton, D., Holmes, T. F., and Maatsch, J. L. | North America | 1977 | American journal of obstetrics and gynecology |
| G31 | Ethical considerations | Implementation and utilization of gynecological teaching associate and male urogenital teaching associate programs: a scoping review. | Hopkins, Holly and Weaks, Chelsea and Napier, Elise | Europe | 2021 | Advances in simulation |
| G32 | Ethical considerations | The association of standardized patient educators (ASPE) gynecological teaching associate (GTA) and male urogenital teaching associate (MUTA) standards of best practice. | Hopkins, Holly and Weaks, Chelsea and Webster, Tim and Elcin, Melih | Europe | 2021 | Advances in Simulation |
| G33 | Ethical considerations Standardisation | Simulation Training Impacts Student Confidence and Knowledge for Breast and Pelvic Examination | Jain, S. and Fox, K. and Van den Berg, P. and Hill, A. and Nilsen, S. and Olson, G. and Karnath, B. and Frye, A. and Szauter, K. | North America | 2014 | Medical Science Educator |
| G34 | Educational outcomes Standardisation | A qualitative study of the impact and acceptability of gynaecological teaching associates | Janjua A. and Burgess L. and Clark T.J. | Europe | 2017 | BJOG: An International Journal of Obstetrics and Gynaecology |
| G35 | Finance | The CEAT Study: Cost Effective Analysis of Teaching pelvic examination skills using gynaecology teaching associates (GTAs) compared with manikin models | Janjua A. and Roberts T. and Clark T.J. | Europe | 2017 | BJOG: An International Journal of Obstetrics and Gynaecology |
| G36 | Educational outcomes Finance Standardisation | The effectiveness of gynaecology teaching associates in teaching pelvic examination to medical students: a randomised controlled trial. | Janjua, Aisha and Smith, P and Chu, J and Raut, N and Malick, S and Gallos, I and Singh, R and Irani, S and Gupta, J K and Parle, J and Clark, T J | Europe | 2017 | European journal of obstetrics, gynecology, and reproductive biology |
| G37 | Educational outcomes Finance | A cross-sectional study on teaching pelvic examination in medical schools in the UK (the COTES study). | Janjua, Aisha and Smith, Paul and Clark, T Justin | Europe | 2018 | Journal of obstetrics and gynaecology : the journal of the Institute of Obstetrics and Gynaecology |
| G38 | Educational outcomes Ethical considerations | Patient involvement in teaching and assessing intimate examination skills: a systematic review. | Jha, V., Setna, Z., Al‐Hity, A., Quinton, N.D. and Roberts, T.E., | Europe | 2010 | Medical Education |
| G39 | Ethical considerations Standardisation | Teaching pelvic examination to second-year medical students using programmed patients. | Johnson, G.H., Brown, T.C., Stenchever, M.A., Gabert, H.A., Poulson, A.M. and Warenski, J.C. | North America | 1975 | American journal of obstetrics and gynecology |
| G40 | Standardisation | Pelvic examination teaching: linking medical student professionalism and clinical competence. | Kamemoto, Lori E and Kane, Kathleen O and Frattarelli, LeighAnn C | North America | 2003 | Hawaii medical journal |
| G41 | Finance | Utility of Gynecological Teaching Associates. | Kelly, Katherine and Wilder, Lauren and Bastin, Jessica and Lane-Cordova, Abbi and Cai, Bo and Cook, James | North America | 2023 | Cureus |
| G42 | Finance Standardisation | Evolution of the Gynecology Teaching Associate: an education specialist. | Kretzschmar, R M | North America | 1978 | American journal of obstetrics and gynecology |
| G43 | Educational outcomes | Simulation training for pelvic examination: A systematic review. | Le Lous, Maela and Dion, Ludivine and Le Ray, Camille | Europe | 2023 | Journal of gynecology obstetrics and human reproduction |
| G44 | Finance Standardisation | Oocyte donors as gynecologic teaching associates. | Legro, R S and Gnatuk, C L and Kunselman, A R and Cain, J | North America | 1999 | Obstetrics and gynecology |
| G45 | Educational outcomes | The professional patient session as a technique for teaching the gynecological examination to nurse practitioner students. | Lehrer, S.S. | North America | 1980 | The Journal of nursing education |
| G46 | Educational outcomes Finance | A follow-up study of patient-instructors who teach the pelvic examination. | Livingstone, R A and Moodie, P F and Ostrow, D N | North America | 1980 | Journal of medical education |
| G47 | Educational outcomes Ethical considerations Standardisation | Professional patient-instructors in the teaching of the pelvic examination. | Livingstone, R A and Ostrow, D N | North America | 1978 | American journal of obstetrics and gynecology |
| G48 | Standardisation | "There is no standard vulva": Sanitized vs. contextualized instruction of hands-on medical skills. | MacFife, Bex., Adamo, G., Adamo, Ahmed, S., Ahmed, Bates, J., Ellaway, R.H., Bates, Bates, J., Schrewe, B., Ella. | North America | 2022 | Social Science & Medicine |
| G49 | Educational outcomes Standardisation | Genital examination training: assessing the effectiveness of an integrated female and male teaching programme. | McBain, L., Pullon, S., Garrett, S. and Hoare, K. | Oceania | 2016 | BMC Medical Education |
| G50 | Educational outcomes Standardisation | The Gynecological Teaching Associates program. | Muggah, H F and Staseson, S | North America | 1988 | The Canadian nurse |
| G51 | Educational outcomes Finance | Use of professional patients in teaching pelvic examinations. | Nelson, L H | North America | 1978 | Obstetrics and gynecology |
| G52 | Finance | Comprehensive pelvic muscle assessment: Developing and testing a dual e-Learning and simulation-based training program | Newman, D.K. and Lowder, J.L. and Meister, M. and Low, L.K. and Fitzgerald, C.M. and Fok, C.S. and Geynisman-Tan, J. and Lukacz, E.S. and Markland, A. and Putnam, S. and Rudser, K. and Smith, A.L. and Miller, J.M. | North America | 2023 | Neurourology and Urodynamics |
| G53 | Educational outcomes Standardisation | Teaching medical students to perform the often-dreaded exam. | Pamela Good | North America | 2020 | Association of American Medical College |
| G54 | Standardisation (Table 1) | Fitting contraceptive diaphragms: Can laywomen provide quality training for doctors? | Pickard, S., Baraitser, P., Rymer, J. and Piper, J. | Europe | 2003 | BMJ |
| G55 | Educational outcomes Standardisation | Can gynaecology teaching associates provide high quality effective training for medical students in the United Kingdom? Comparative study. | Pickard, Sally and Baraitser, Paula and Rymer, Janice and Piper, Johanna | Europe | 2003 | BMJ (Clinical research ed.) |
| G56 | Educational outcomes Standardisation | Students' and physicians' evaluations of gynecologic teaching associate program. | Plauche, Warren C and Baugniet-Nebrija, Wendy | North America | 1985 | Journal of Medical Education |
| G57 | Finance (Table 2) Ethical considerations (Table 2) | The quandary of the sacred vagina: Exploring the value of gynaecological teaching associates. | Posner, Glenn D and Baecher-Lind, L. E., Chang, K., Blanco, M. A., Baecher-Lind, Issenberg, S. B., Scalese, R. J., Is... | North America | 2015 | Medical Education |
| G58 | Educational outcomes Finance | Evaluating pelvic examination training: Does faculty involvement make a difference? A randomized controlled trial. | Pradhan, Archana and Ebert, Gary and Brug, Pamela and Swee, David and Ananth, Cande V and Abraham, S., Abraham, Barley, G., Fischer, J., Dwinnell, B., White, K., Barley, Beckmann, C. R. B... | North America | 2010 | Teaching and Learning in Medicine |
| G59 | Ethical considerations Standardisation | Women teaching women's health: issues in the establishment of a clinical teaching associate program for the well woman check. | Robertson, Kathryn and Hegarty, Kelsey and O'Connor, Vivienne and Gunn, Jane | Oceania | 2003 | Women & health |
| G60 | Educational outcomes Finance Standardisation | Use of male and female professional patient teams in teaching physical examination of the genitalia. | Rochelson, B L and Baker, D A and Mann, W J and Monheit, A G and Stone, M L | North America | 1985 | The Journal of reproductive medicine |
| G61 | Educational outcomes | Medical student confidence when training for a female genitourinary exam using models and standardized patients. | Rutledge, M., Link, K., Zapata, I. and Carter, S | North America | 2022 | Journal of Obstetrics and Gynaecology Research |
| G62 | Educational outcomes Standardisation | Success of the first gynecological teaching associate program in Turkey. | Sarmasoglu, S., Dinc, L., Elcin, M., Celik, G.H.T. and Polonko, I | Europe | 2016 | Clinical simulation in nursing |
| G63 | Standardisation | Pelvic examination skills training with genital teaching associates and a pelvic simulator: does sequence matter?. | Seago, Brenda L and Ketchum, Jessica M and Willett, Rita M | North America | 2012 | Simulation in healthcare |
| G64 | Educational outcomes (Table 1 & 2) | Acquisition of pelvic examination skills: Evaluation of student feelings about a surrogate patient program. | Shain, R. N and Crouch, S. H and Weinberg, P. C | Europe | 1983 | Journal of Psychosomatic Obstetrics & Gynecology |
| G65 | Educational outcomes | Evaluation of the gynecology teaching associate versus pelvic model approach to teaching pelvic examination. | Shain, Rochelle N and Crouch, Susan H and Weinberg, Paul C | North America | 1982 | Journal of Medical Education |
| G66 | Educational outcomes | Learning pelvic examination with professional patients. | Shrestha S., Wijma B., Swahnberg k. and Siwe, K. | Asia | 2010 | JNMA; journal of the Nepal Medical Association |
| G67 | Ethical considerations | Including gynecological teaching associates' perspectives in women's health exams: Lessons for improved communication practices. | Silverman, Rachel E and Araujo, Meagan and Nicholson, Abby and Bell, S., Bell, Boston Women's Health Collective, Butler, J., Butler, Ehrenreigh, B., English, D. | North America | 2012 | Health Communication |
| G68 | Ethical considerations | "Teaching those who teach to have a voice: the history and current practices of Gynecological Teaching Associates." | Silverman, Rachel E. | North America | 2014 | Journal of Medicine and the Person |
| G69 | Educational outcomes | A stronger and clearer perception of self'. Women's experience of being professional patients in teaching the pelvic examination: a qualitative study. | Siwe, K and Wijma, B and Bertero, C | Europe | 2006 | BJOG : an international journal of obstetrics and gynaecology |
| G70 | Educational outcomes | Unexpected enlightening of a "female world". Male medical students' experiences of learning and performing the first pelvic examination. | Siwe, Karin and Bertero, Carina and Wijma, Barbro | Europe | 2012 | Sexual & reproductive healthcare : official journal of the Swedish Association of Midwives |
| G71 | Educational outcomes | Performing the first pelvic examination: female medical students' transition to examiners. | Siwe, Karin and Wijma, Barbro and Silen, Charlotte and Bertero, Carina | Europe | 2007 | Patient Education and Counseling |
| G72 | Finance Ethical considerations | Medical students learning the pelvic examination: Comparison of outcome in terms of skills between a professional patient and a clinical patient model. | Siwe, Karin and Wijma, Klaas and Stjernquist, Martin and Wijma, Barbro and Abraham, S., Chapman, M., Taylor, A., McBride, A., Boyd, C., Abraham, Abraham, S., Abraham, A | Europe | 2007 | Patient Education and Counseling |
| G73 | Educational outcomes Standardisation | The effectiveness of gynaecological teaching associates in teaching pelvic examination: A systematic review and meta-analysis. | Smith, Paul P and Choudhury, Shelina and Clark, T. Justin and Abraham, S., Abraham, Beckmann, C. R., Spellacy, W. N., Yonke, A., Barzansky, B., Cunningham, R. | Europe | 2015 | Medical Education |
| G74 | Ethical considerations | Gynaecological teaching associates are not the answer | Sohaib, S.M | Europe | 2004 | BMJ |
| G75 | Educational outcomes Ethical considerations | Learning the pelvic exaamination by clerkship medical students: evaluating skills by standardized patient model. | Sultana, S., Khan, M.N.A., Sharif, S., Khan, N.M. and Sadia, S.N. | Asia | 2015 | Pakistan Armed Forces Medical Journal |
| G76 | Recruitment (Table 2) | UK‑GTA: Gynaecological Teaching Associates. | UK-GTA Ltd | Europe | 2018 | UK-GTA |
| G77 | Ethical considerations Standardisation | “It’s the Knowledge That Puts You in Control” The Embodied Labor of Gynecological Educators. | Underman, K. | North America | 2011 | Gender & Society |
| G78 | Educational outcomes Ethical considerations | Playing doctor: Simulation in medical school as affective practice. | Underman, Kelly and Ahmed, S., Ahmed, Becker, H., et al., Becker, Beckmann, C.R.B., et al., Beckmann, Bourdieu, P. | North America | 2015 | Social Science & Medicine |
| G79 | Standardisation | Training of the gynaecological examination in The Netherlands. | Van Ravesteijn, Hiske and Hageraats, Emer and Rethans, Jan-Joost | Europe | 2007 | Medical Teacher |
| G80 | Educational outcomes Finance Standardisation | The effects of two methods of pelvic examination instruction on student performance and anxiety. | Vontver, L and Irby, D and Rakestraw, P and Haddock, M and Prince, E and Stenchever, M | North America | 1980 | Journal of Medical Education |
| G81 | Educational outcomes Ethical considerations | Teaching pelvic examination technique using professional patients: a controlled study evaluating students' skills. | Wanggren, Kjell and Fianu Jonassen, Aino and Andersson, Sonja and Pettersson, Gunilla and Gemzell-Danielsson, Kristina | Europe | 2010 | Acta obstetricia et gynecologica Scandinavica |
| G82 | Educational outcomes Ethical considerations Standardisation | Teaching medical students gynaecological examination using professional patients-evaluation of students' skills and feelings. | Wanggren, Kjell and Pettersson, Gunilla and Csemiczky, Gyorgy and Gemzell-Danielsson, Kristina | Europe | 2005 | Medical Teacher |
| G83 | Ethical considerations | At Your Cervix: A'magine and Renee Bergan | Williams, K. | North America | 2023 | IEEE Women in Engineering Magazine |
