# Supplementary material for: Gynaecology Teaching Associates in Medical Education—A Scoping Review
Source: Clin Teach. 2026 Jun 10;23(4):e70460. doi: 10.1111/tct.70460 (PMC13250820; doi:10.1111/tct.70460)
Supplement: Supplementary file 3 — Data S3: PRISMA Flow Diagram. [file TCT-23-e70460-s002.docx]

Search Strategy

Scoping Review: Gynaecology Teaching Associates

# Medline

| **Ovid MEDLINE(R) and In-Process, In-Data-Review & Other Non-Indexed Citations 1946 to July 18, 2025**  **Accessed via ovid.com \| Run Date: 21^st^ July 2025** | | |
| --- | --- | --- |
| **Line#** | **Search String** | **No.** |
| 1 | gynecology/ed OR gynecological examination/ OR genitalia, female/ OR uterus/ OR cervix uteri/ OR vagina/ OR vulva/ OR adnexa uteri/ OR ovary/ OR pelvis/ OR (pelvic exam* OR speculum OR bimanual OR vaginal exam*).ti,ab,kf. | 239611 |
| 2 | (GTA? OR laywom#n OR professional patient? OR patient instructor? OR gyn?e* teaching associate?).ti,ab,kf. | 16919 |
| 3 | 1 AND 2 | 101 |
| 4 | Limit to English Language | 97 |

# Embase

| **EMBASE 1974 – 2025 July 18**  **Accessed via ovid.com \| Run Date: 21^st^ July 2025** | | |
| --- | --- | --- |
| **Line#** | **Search String** | **No.** |
| 1 | gynecology/ OR gynecological examination/ OR female genital system/ OR uterus/ OR uterine cervix/ OR vagina/ OR vulva/ OR pelvis/OR pelvic examination/ OR genital system examination/ OR vaginal speculum/ OR (bimanual OR vaginal exam*).ti,ab,kw. | 331972 |
| 2 | (GTA? OR laywom#n OR professional patient? OR patient instructor? OR gyn?e* teaching associate?).ti,ab,kw. | 2916 |
| 3 | 1 AND 2 | 80 |
| 4 | Limit to English Language | 76 |

# PsycINFO

| **APA PsychInfo 1806 to July 2025 Week 2**  **Accessed via ovid.com \| Run Date: 21^st^ July 2025** | | |
| --- | --- | --- |
| **Line#** | **Search String** | **No.** |
| 1 | gynecology/ OR physical examination/ OR female genitalia/ OR uterus/ OR cervix / OR vagina/ OR (pelvic exam* OR speculum OR bimanual OR vaginal exam* OR vulva OR pelvis).ti,ab,id. | 9748 |
| 2 | (GTA? OR laywom#n OR professional patient? OR patient instructor? OR gyn?e* teaching associate?).ti,ab,id | 738 |
| 3 | 1 AND 2 | 21 |
| 4 | Limit to English Language | 20 |

# Scopus

| **Scopus**  **Accessed via scopus.com \| Run Date: 21^st^ July 2025** | | |
| --- | --- | --- |
| **Line#** | **Search String** | **No.** |
| 1 | TITLE-ABS-KEY(gynecology OR gynaecology OR “gynecological exam” OR “gynaecological exam” OR “gynecological examination” OR “gynaecological examination” OR “gynecology exam” OR “gynaecology exam” OR “gynecology examination” OR “gynaecology examination” OR “female genital” OR “female genitals” OR uterus OR “uterine cervix” OR vagina OR vulva OR adnexa OR ovary OR pelvis OR “pelvic exam” OR “pelvic examination” OR “genital system exam” OR “genital system examination” OR speculum OR bimanual OR “vaginal exam” OR “vaginal examination”) |  |
| 2 | TITLE-ABS-KEY(GTA OR GTAs OR laywoman OR laywomen OR “professional patient” OR “professional patients” OR “patient instructor” OR “patient instructors” OR “gynae teaching associate” OR “gynae teaching associates”  OR “gynae teaching assistant” OR “gynae teaching assistants” OR “gyne teaching associate” OR “gyne teaching associates” OR “gyne teaching assistant” OR “gyne teaching assistants” OR “gynaecology teaching associate” OR “gynaecology teaching associates” OR “gynaecology teaching assistant” OR “gynaecology teaching assistants” OR “gynecology teaching associate” OR “gynecology teaching associates” OR “gynecology teaching assistant” OR “gynecology teaching assistants”) |  |
| 3 | 1 AND 2 | 599 |
| 4 | Limit to English Language | 567 |

# CINAHL

| **CINAHL (Cumulative Index to Nursing and Allied Health Literature)**  **Accessed via EBSCOHost.com \| Run Date: 21^st^ July 2025** | | |
| --- | --- | --- |
| **Line#** | **Search String** | **No.** |
| 1 | XB(gynecology OR gynaecology OR “gynecological exam” OR “gynaecological exam” OR “gynecological examination” OR “gynaecological examination” OR “gynecology exam” OR “gynaecology exam” OR “gynecology examination” OR “gynaecology examination” OR “female genital” OR “female genitals” OR uterus OR “uterine cervix” OR vagina OR vulva OR adnexa OR ovary OR pelvis OR “pelvic exam” OR “pelvic examination” OR “genital system exam” OR “genital system examination” OR speculum OR bimanual OR “vaginal exam” OR “vaginal examination”) |  |
| 2 | XB(GTA OR GTAs OR laywoman OR laywomen OR “professional patient” OR “professional patients” OR “patient instructor” OR “patient instructors” OR “gynae teaching associate” OR “gynae teaching associates”  OR “gynae teaching assistant” OR “gynae teaching assistants” OR “gyne teaching associate” OR “gyne teaching associates” OR “gyne teaching assistant” OR “gyne teaching assistants” OR “gynaecology teaching associate” OR “gynaecology teaching associates” OR “gynaecology teaching assistant” OR “gynaecology teaching assistants” OR “gynecology teaching associate” OR “gynecology teaching associates” OR “gynecology teaching assistant” OR “gynecology teaching assistants”) |  |
| 3 | 1 AND 2 | 23 |
| 4 | Limit to English Language | 23 |

# ERIC

| **ERIC (Education Resource Information Center)**  **Accessed via EBSCOHost.com \| Run Date: 21^st^ July 2025** | | |
| --- | --- | --- |
| **Line#** | **Search String** | **No.** |
| 1 | TX(gynecology OR gynaecology OR “gynecological exam” OR “gynaecological exam” OR “gynecological examination” OR “gynaecological examination” OR “gynecology exam” OR “gynaecology exam” OR “gynecology examination” OR “gynaecology examination” OR “female genital” OR “female genitals” OR uterus OR “uterine cervix” OR vagina OR vulva OR adnexa OR ovary OR pelvis OR “pelvic exam” OR “pelvic examination” OR “genital system exam” OR “genital system examination” OR speculum OR bimanual OR “vaginal exam” OR “vaginal examination”) |  |
| 2 | TX(GTA OR GTAs OR laywoman OR laywomen OR “professional patient” OR “professional patients” OR “patient instructor” OR “patient instructors” OR “gynae teaching associate” OR “gynae teaching associates”  OR “gynae teaching assistant” OR “gynae teaching assistants” OR “gyne teaching associate” OR “gyne teaching associates” OR “gyne teaching assistant” OR “gyne teaching assistants” OR “gynaecology teaching associate” OR “gynaecology teaching associates” OR “gynaecology teaching assistant” OR “gynaecology teaching assistants” OR “gynecology teaching associate” OR “gynecology teaching associates” OR “gynecology teaching assistant” OR “gynecology teaching assistants”) |  |
| 3 | 1 AND 2 | 6 |
| 4 | Limit to English Language | 6 |
