# Supplementary material for: Gynaecology Teaching Associates in Medical Education—A Scoping Review
Source: Clin Teach. 2026 Jun 10;23(4):e70460. doi: 10.1111/tct.70460 (PMC13250820; doi:10.1111/tct.70460)
Supplement: Supplementary file 4 — Data S4: Summary of Included Studies. [file TCT-23-e70460-s004.docx]

**Identification of studies via other methods**

**Identification of studies via databases and registers**

Records identified from:

**Citation searching (Total =458)**

Backward citations (n = 73)

Forward citations (n = 385)

**Targeted searching (Total = 14)** Website (n = 3)

News Article (n = 2)

Journal Article (n = 8)

Thesis (n = 1)

Records removed *before screening*:

Duplicate records removed

(n = 192)

Records identified from:

**Databases (Total = 789)**

Medline (n = 97)

Embase (n = 76)

PsycInfo (n = 20)

Scopus (n = 567)

ERIC (n = 6)

**Identification**

Records screened

(n = 597)

Records excluded

(n = 505)

Reports not retrieved

(n = 21)

Reports sought for retrieval

(n = 69)

Reports sought for retrieval

(n = 92)

Reports not retrieved

(n = 6)

**Screening**

Reports assessed for eligibility

(n = 48)

Reports excluded:

Publication Type (n = 3)

Content (n = 21)

Reports assessed for eligibility

(n = 86)

Reports excluded:

Publication Type (n =13)

Content (n = 14)

Reports of included studies

**(n = 83)**

**Included**

Source: Page MJ, et al. BMJ 2021;372:n71. doi: 10.1136/bmj.n71.

This work is licensed under CC BY 4.0. To view a copy of this license, visit <https://creativecommons.org/licenses/by/4.0/>
